# Can Ultrasound-Guided Continuous Paravertebral Block Reduce the Incidence of Chronic Postsurgical Pain in Patients with Thoracoscopic Lung Cancer Surgery? A Randomized Controlled Trial

**DOI:** 10.1155/2023/6433494

**Published:** 2023-11-09

**Authors:** Wei Ran, Huan Luo, Zhiqiao Wang, Yonggang Hao, Ning Liang, Ping Li, Xia Yin, Jin Gao

**Affiliations:** Department of Anesthesiology, The First Affiliated Hospital of Chongqing Medical University, Chongqing 400016, China

## Abstract

**Background:**

Thoracoscopic lung cancer surgery is accompanied by severe pain. Both continuous paravertebral block (CPVB) and continuous wound infiltration (CWI) are widely used for perioperative analgesia in thoracoscopic surgery. However, the effects of these different methods on chronic postsurgical pain (CPSP) are still unknown. *Patients and Methods*. This prospective randomized controlled trial assessed the eligibility of 113 patients. Ninety-seven patients who met the inclusion criteria were randomly divided into a CPVB group and a CWI group, and 80 patients were analyzed in the final study. The primary outcome measures were the incidence and intensity of chronic postsurgical pain (CPSP) at 3, 6, and 9 months after surgery. The secondary outcome measures were the numerical rating scale (NRS) score of rest and activity at 12, 18, and 24 hours and on the 2^nd^, 3^rd^, and 7^th^ days postoperatively; the Barthel Activities of Daily Living (ADL) score of activity levels on the 1^st^, 2^nd^, 3^rd^, and 7^th^ days postoperatively; and the long-term quality of the life score at 3, 6, and 9 months postoperatively.

**Results:**

The incidence of chronic postsurgical pain in the CWI group was significantly higher than that in the CPVB group at 3, 6, and 9 months after surgery (all *P* < 0.05). The intensity of chronic postsurgical pain was significantly decreased in the CPVB group at 3, 6, and 9 months after surgery (*P* < 0.05). NRS-R and NRS-A scores were significantly decreased in the CPVB group within the first week after thoracoscopic surgery (*P* < 0.001). ADL scores were increased in the CPVB group within 3 days postoperatively. However, there were no differences in the ADL score on the 7^th^ postoperative day or the long-term quality of the life score at 3, 6, and 9 months postoperatively.

**Conclusion:**

Continuous ultrasound-guided paravertebral block reduced the intensity of acute pain within 7 days postoperatively and reduced the incidence of chronic pain at 3, 6, and 9 months after surgery, but there was no significant advantage in long-term quality of life. This trial is registered with ChiCTR2000038505.

## 1. Introduction

Lung cancer is the most common malignant tumor in the world, and surgery has been the main treatment option until now [1]. However, some patients may also experience chronic postsurgical pain (CPSP) due to surgical incisions, expander compression, drainage tube depression, and rib and nerve injury [2]. It has been reported that the incidence of CPSP in patients after lung cancer surgery is 25%–75% [3]. The definition of chronic postsurgical pain (CPSP) in the International Association for the Study of Pain (IPSP) is pain that develops or increases in intensity after a surgical procedure or a tissue injury and persists beyond the healing process, i.e., at least 3 months after surgery or tissue trauma. The pain is either localized to the surgical field or the area of injury, projected to the innervation territory of a nerve situated in this area, or referred to a dermatome [4]. Due to the psychological distress and disability caused by CPSP, it has a great negative impact on quality of life. Therefore, it has become an important problem for patients who receive lung cancer surgery.

Multimodal analgesia strategies are recommended for the management of pain [5]. Nerve block and local anesthetic analgesia are common techniques combined with opioids that aim to reduce short-term pain in thoracic surgery [6, 7]. Previous guidelines pointed out that the European Society of Anesthesia recommends continuous wound infiltration analgesia for postoperative pain management after lung cancer surgery. Because it is an effective, low-cost, proven, and safe analgesic technique, it is also easy to perform [8, 9]. However, with the development of ultrasound technology, paravertebral block has gradually become a new choice for perioperative analgesia in thoracoscopic surgery, with a high success rate and a low rate of serious complications [10, 11].

Both analgesic techniques have advantages, but the comparative impact of these techniques on CPSP functional recovery and quality of life after patients receive thoracoscopic surgery has not been explored to date. Therefore, our study aims to compare the efficacy of ultrasound-guided continuous paravertebral block (CPVB) and continuous wound infiltration (CWI) on the incidence and intensity of CPSP and long-term quality of life after thoracoscopic lung cancer surgery.

## 2. Methods

### 2.1. The Study Design and Participants

This prospective randomized clinical trial was conducted after obtaining approval from the Chinese Clinical Trial Registry (http://www.chictr.org.cn/showproj.aspx?proj=61825ChiCTR2000038505) and approved by the Ethics Committee of the First Affiliated Hospital of Chongqing Medical University (IRB: 2020-407). This study was performed at the First Affiliated Hospital of Chongqing Medical University in accordance with the Consolidated Standards of Reporting Trials Statement for Reporting Trials August 2020 to January 2021. Written informed consent was obtained from each patient before recruitment.

#### 2.1.1. Inclusion Criteria

Patients with lung cancer undergoing elective thoracoscopic surgery, age over 18 years, BMI 18–40 kg/m2, and American Society of Anesthesiologists (ASAs) physical status II-III were enrolled in this study.

#### 2.1.2. Inclusion Criteria for Chronic Postsurgical Pain

Pain that occurs or increases after surgery or tissue injury lasts at least three months and is limited to the surgical or injury site.

#### 2.1.3. Exclusion Criteria

Contraindications to CPVB included allergies to local anesthetics, infection, or coagulopathy; allergic constitution; severe cardiovascular or hepatorenal insufficiency; coagulation system disease; morbid obesity (BMI >40 kg/m^2^); history of chronic pain; presence of neuropsychiatric diseases; and inability to comply with the study protocol for any reason. Other causes of pain, including infection, malignancy, and preexisting pain, were excluded.

#### 2.1.4. Randomization and Blinding

The computer random number method generated a total of 113 random numbers (ranging from 1 to 113). The first 56 numbers were assigned to the CPVB group, and the remaining 57 digits were assigned to the CWI group. Patients ranked the numbers sequentially according to the experiment, and statistical analysis was finally performed based on the grouped information. Surgeries were performed by the same group of surgeons. The patients, data collector, and care team were blinded, while the anesthesiologists and surgeons were unblinded participants who were not otherwise involved in the study.

### 2.2. Anesthesia Procedure

After patient arrival at the operation room, an intravenous sodium lactate Ringer's solution infusion was initiated and a radial artery catheter was placed. Standard monitoring, including evaluation of pulse oxygen saturation (SpO_2_), end-tidal carbon dioxide partial pressure (PETCO_2_), bispectral index monitoring (BIS), 3-lead electrocardiography, body temperature (T), and urine output, was performed. Anesthesia and operations were performed after a safety check. Midazolam (0.04 g/kg), sufentanil (0.5 g/kg), propofol (1.5 mg/kg), and vecuronium (0.1 mg/kg) were administered for induction. Intubation was performed using a double-lumen endobronchial tube, and the ipsilateral lung was deflated during the procedure. The lungs were ventilated with VC-IPPV set at Vt 5–8 mL/kg, I/E ratio of 1 : 2, and a respiratory rate sufficient to maintain PETCO_2_ at 35–45 mmHg. The target BIS value was maintained at 40–60. General anesthesia was maintained by infusion of propofol, sufentanil, and sevoflurane. Small doses of noradrenaline were used to maintain the invasive arterial blood pressure (IBP) and the heart rate (HR) at a normal stage.

### 2.3. Ultrasound-Guided CPVB

**Figure 1:**
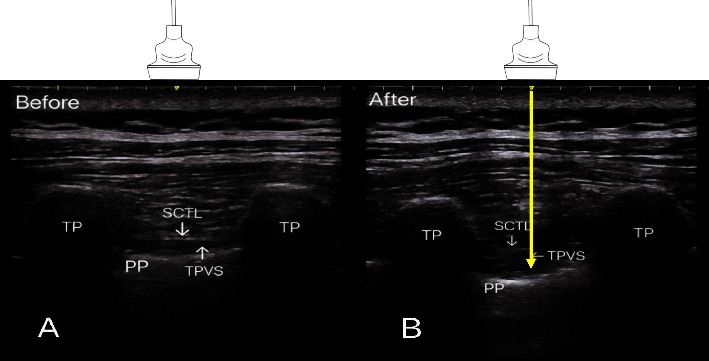
The nerve block needle reached the position through the out-of-plane technique (A); after confirming negative aspiration, ropivacaine was injected (B). TP, transverse process; PP, parietal pleural; SCTL, superior costotransverse ligament; TPVS, thoracic paravertebral space; probe selection: low-frequency linear array probe; yellow arrow: puncture approach.

In the CPVB group, ultrasound-guided CPVB was performed with the patient in the lateral position by the same anesthesiologist at the end of the operation. After identifying the paravertebral space of the proposed level of the intercostal skin incision, a 20-gauge catheter (B. Braun Melsungen AG, 34209 Melsungen, Germany) was inserted 2-3 cm into the paravertebral space by using an out-of-plane approach with an 18-gauge Tuohy cannula needle. When there was no blood or cerebrospinal fluid reflux after suction, a bolus of 1-2 ml of saline solution was injected through the PVB catheter to confirm the correct position, and then, 15 ml of 0.33% ropivacaine was given (Figure 1). Within 48 hours, continuous paravertebral patient-controlled analgesia (PCA) was administered (a capacity of 300 mL, including 1% ropivacaine 600 mg, fentanyl 0.5 mg, saline 230 ml; a background dose of 5 ml/h, a bolus dose of 5 ml, and a lock-out time of 45 minutes).

**Figure 2:**
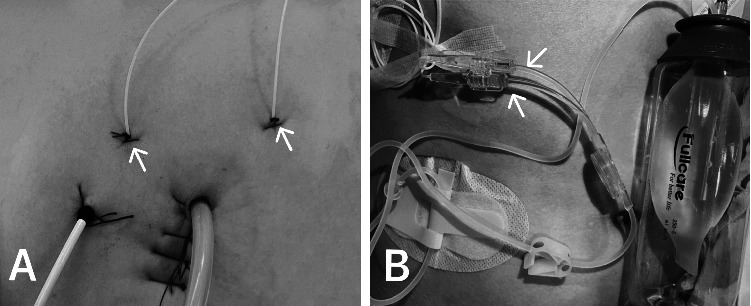
The position of the multiperforated wound catheters (A); the catheters were connected to a disposable postoperative local anesthesia analgesia device (B).

Before skin closure, the same surgeon placed two multiperforated wound catheters (PAINfusor®, Baxter, Maurepas, France) between the serratus anterior and the intercostal external in the CWI group. The catheter was inserted three to four centimeters from the incision and sutured to the skin (Figure 2(A)). Catheter permeability was tested before a bolus injection of 10 mL of 0.33% ropivacaine. Then, the catheters were connected to a disposable postoperative local anesthesia analgesia device (TJPS060-2-250-5) containing 300 ml of 0.2% ropivacaine and a background dose of 5 mL/h (Figure 2(B)). The catheters were removed after 48 hours by the surgical team.

After the surgery, acupuncture hypoalgesia and hypothermia tests were used to confirm whether the dermatomal level of sensory blockade had reached the fourth thoracic vertebra by 4 hours postoperatively, and patients with failed blockade were excluded. For rescue analgesia, dezocine (10 mg) was given intravenously immediately when NRS exceeded 4 in the resting state.

## 3. Outcome Measures

### 3.1. The Main Outcome Measures

The incidence of CPSP was assessed by the number of positive cases of CPSP (NRS ≥ 1 and 3 months postoperatively). The intensity of CPSP can be indirectly assessed by the scores of the effects of pain on daily life and the level of pain treatment measures [12]. The patient's initial pain the day after surgery was referred to as postoperative pain. The pain felt in the first week after surgery was referred to as persistent postoperative pain. Finally, chronic pain was defined as lasting more than three months after surgery.

The effect of pain on daily life had four grades: grade A (no effect), grade B (mild effect), grade C (moderate effect), and grade D (severe effect).

The level of pain treatment measures had four grades: grade A (no treatment measures), grade B (rest or reduced activity), grade C (self-medication), and grade D (treatment at a hospital).

### 3.2. The Secondary Outcome Measures

Secondary outcome measures were as follows: (1) general information of the patients; (2) the NRS score for rest and activity at 12, 18, and 24 hours as well as 2, 3, and 7 days postoperatively; (3) the Barthel Activities of Daily Living (ADL) score for the activity level at 1^st^, 2^nd^, 3^rd^, and 7^th^ day postoperatively: this scale comprises 10 basic daily activities (bowel control, bladder control, feeding, toileting, bathing, dressing, grooming, walking, stair climbing, and chair-to-bed/bed-to-chair transfer), with each item scored as 0 = need complete help, 1 = need some help, or 2 = need no help [13]. (4) The long-term quality of the life score at 3, 6, and 9 months postoperatively: long-term quality of life was assessed by the 12-item Short-Form Health Survey (SF-12), which is a 12-item (domain) questionnaire. Two summary components were constructed to summarize the physical and mental components (PCS and MCS, respectively). The scale was explained to the patients by a research doctor who was unaware of the group allocation. (5) Postoperative-related data included additional analgesic requirement, patient satisfaction with postoperative analgesia (if patients had an NRS score of <2, the postoperative analgesia was considered satisfactory), adverse effects after surgery, length of ICU stay, time of chest tube removal, time of tracheal tube removal, and length of hospital stay.

The patients were divided into two groups according to whether the NRS-R score was ≥2 on the first day after the operation. The incidence of CPSP in the two groups was followed up at 3, 6, and 9 months after surgery.

All patients were interviewed by telephone at 3, 6, and 9 months after surgery to collect postoperative data.

## 4. Sample Size Calculation

Sample size calculation was performed using PASS 15.0 (Stata Corp. LP, College Station, Texas, USA), which relied on the chronic pain incidence at 3 months after surgery. According to our preliminary results of 38 cases (19 cases in the CPVB group and 19 cases in the CWI group), we found that the incidence of CPSP at 3 months was approximately 10.5% in the CPVB group and 42.1% in the CWI group. Using a two-proportion test with a power of 90% (*α* = 0.05), the minimal sample size needed to be 39 patients in each group to detect differences between the two groups. Considering a 20% dropout rate, we ultimately included a total of 113 patients.

## 5. Statistical Analysis

All statistical analyses were performed using the Statistical Package for the Social Sciences version 26.0 (SPSS Inc., Chicago, USA). Parametric variables were compared by an independent *t* test and are reported as the mean (standard deviation). Nonparametric variables were compared using the Mann‒Whitney *U* test and are reported as medians (interquartile ranges (IQRs)). Categorical variables are listed as a ratio or as the numbers and percentage, and the differences between two groups were analyzed by Fisher's exact test or the chi-squared test for trends. All comparisons were two-tailed, and a *P* value <0.05 was considered statistically significant.

## 6. Results

### 6.1. Patient Characteristics

**Figure 3:**
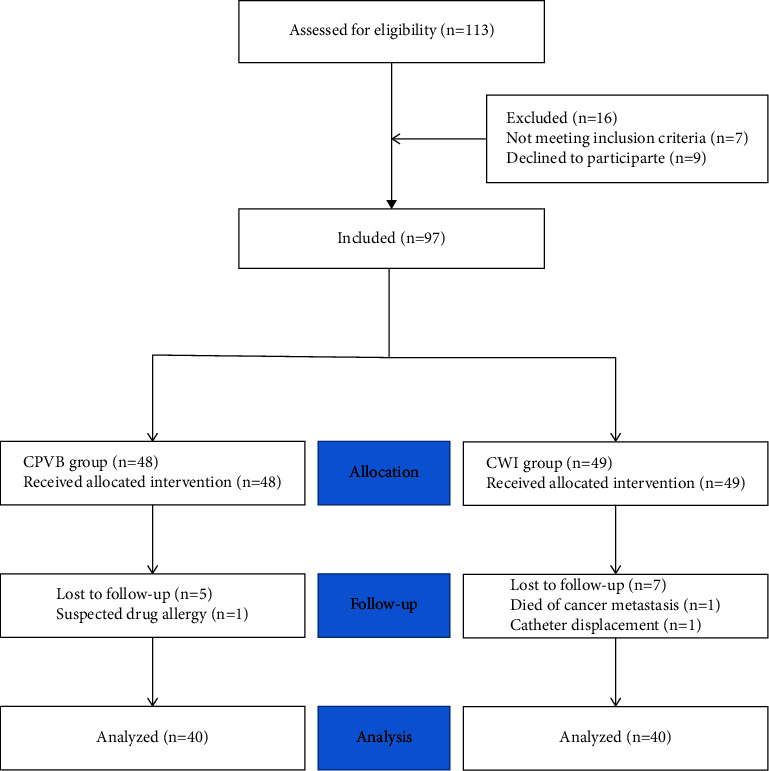
Patient flow diagram. CPVB, continuous paravertebral block; CWI, continuous wound infiltration.

Between August 2020 and January 2021, 113 patients with lung cancer undergoing elective thoracoscopic surgery were enrolled in our study. Seven patients were excluded for the following reasons: history of chronic pain (*n* = 4), neuropsychiatric diseases (*n* = 1), morbid obesity (BMI >40 kg/m^2^) (*n* = 1), and conversion to thoracotomy (*n* = 1). Furthermore, nine patients refused to participate. Seventeen patients were excluded from the final analysis because of mechanical obstruction of the PCA device (*n* = 2), catheter displacement (*n* = 1), death due to cancer metastasis (*n* = 1), suspected drug allergy (*n* = 1), and loss of follow-up (*n* = 12). Consequently, data were analyzed for 40 patients in each group.  Figure 3 shows the Consolidated Standards of Reporting Trials flow diagram. Both groups of patients were comparable in terms of surgical and demographic data (Table 1).

### 6.2. The Incidence and Intensity of CPSP

**Figure 4:**
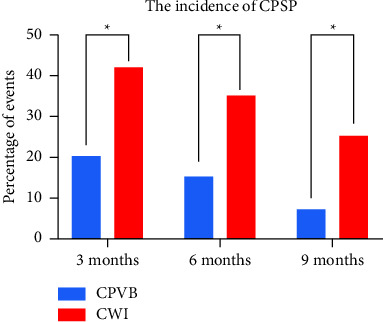
The incidence of CPSP. CPVB, continuous paravertebral block; CWI, continuous wound infiltration; CPSP, chronic postsurgical pain.

The CPSP incidence is shown in Figure 4. The CPSP incidence was significantly higher in the CWI group (42.5% vs. 20% *P*=0.030) than in the CPVB group at the 3^rd^ month postoperatively; the CPSP incidence was significantly higher in the CWI group (35.0% vs. 15.0% *P*=0.039) than in the CPVB group at the 6^th^ month postoperatively, while at the 9^th^ month, the incidence of CPSP was significantly (25.0% vs. 7.5% *P*=0.034) higher in the CWI group than in the CPVB group. The intensity of CPSP, the impact score of pain on daily life (3^rd^ month postoperatively, *P*=0.021; 6^th^ month postoperatively, *P*=0.048; 9^th^ month postoperatively, *P*=0.048), and the pain treatment measures (3^rd^ month postoperatively, *P*=0.014; 6^th^ month postoperatively, *P*=0.030; 9^th^ month postoperatively, *P*=0.041) in the CPVB group were significantly lower than those in the CWI group (Table 2).

### 6.3. Pain Management within 7 Days after Surgery

**Figure 5:**
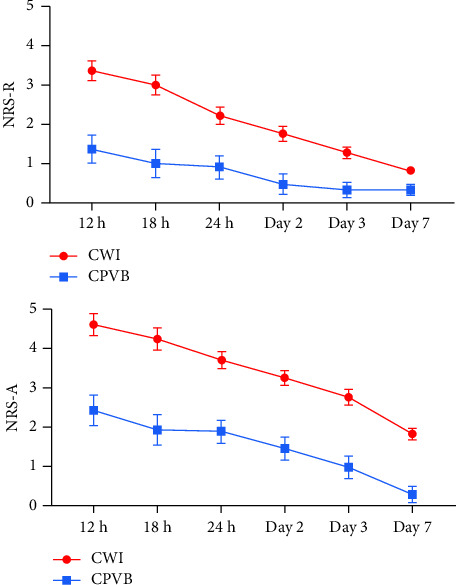
Postoperative pain score during the first week after thoracoscopic surgery (NRS-R: *F* = 41.044 (*P* < 0.001); NRS-A: *F* = 55.290 (*P* < 0.001)). CPVB, continuous paravertebral block; CWI, continuous wound infiltration; NRS-R, numeric rating scale at rest; NRS-A, numeric rating scale at activity.

Acute pain scores at rest are shown in Figure 5. The NRS-A scores of patients in the CPVB group were lower than those in the CWI group at the 6th, 12th, and 18th hours and the 1st, 2nd, 3rd, and 7th days after surgery (the CPVB group vs. the CWI group: *F* = 41.044, *P* < 0.001; *F* = 55.290, *P* < 0.001).

Acute pain scores with activity are shown in Figure 5. At 6, 12, and 18 hours and on the 1^st^, 2^nd^, 3^rd^, and 7^th^ days, the NRS score was significantly lower in the CPVB group than in the CWI group (the CPVB group versus the CWI group: 12 hours postoperatively: 2.43 ± 1.36 versus 4.63 ± 2.11, *P* < 0.001; 18 hours postoperatively: 1.95 ± 1.32 versus 4.25 ± 2.12, *P* < 0.001; 24 hours postoperatively: 1.90 ± 1.13 versus 3.73 ± 1.62, *P* < 0.001; 2 days postoperatively: 1.48 ± 0.96 versus 3.25 ± 1.61, *P* < 0.001; 3 days postoperatively: 0.98 ± 0.92 versus 2.78 ± 1.54, *P* < 0.001; 7 days postoperatively: 0.30 ± 0.61 versus 1.83 ± 1.22, *P* < 0.001).

### 6.4. Barthel ADL Scores within 7 days Postoperatively

The Barthel ADL scores within 7 days postoperatively are shown in Table 3. The Barthel ADL scores were comparable (the CPVB group versus the CWI group: 95.00 [95.00–100.00] versus 95.00 [95.00–100.00], *P*=0.379) between the two groups on Day 7 (the CPVB group versus the CWI group: 75.00 [65.00–80.00] versus 67.50 [60.00–70.00], *P*=0.002), Day 2 (the CPVB group versus the CWI group: 90.00 [80.00–95.00] versus 75.00 [70.00–90.00], *P* < 0.001), and Day 3 (the CPVB group versus the CWI group: 95.00 [90.00–95.00] versus 90.00 [85.00–95.00], *P*=0.005).

### 6.5. Long-Term Quality of the Life Score

The two groups were comparable regarding the SF-12 scores, but there were no differences in long-term quality of life scores at 3, 6, and 9 months postoperatively (Table 4).

### 6.6. The Relationship between Acute Pain Intensity and Chronic Pain Incidence

There were 48 patients with NRS-R scores >2 (Group A) and 32 patients with NRS-R < 2 (Group B) on the first postoperative day. However, there were 20 (41.67%) patients with CPSP in Group A at 3 months, 16 (33.33%) at 6 months, and 11 (22.92%) at 9 months, whereas there were only 5 (15.63%) patients with CPSP in Group B at 3 months, 4 (12.50%) at 6 months, and 2 (6.25%) at 9 months (Table 3). The incidence of CPSP in Group A was increased significantly, which indicated that the acute pain intensity (patients with NRS-R scores >2 on the first day postoperatively) can predict the incidence of CPSP 3 months postoperatively (Table 5).

### 6.7. Postoperative-Related Data

During the 48-hour postoperative period, the CPVB group used significantly less rescue analgesia (2/40 vs. 13/40, *P*=0.002) and had higher analgesia satisfaction (33/40 vs. 15/40, *P*=0.001). The incidence of adverse events such as respiratory depression and urinary retention was similar in the two groups (*P* > 0.050). However, there was a significant reduction in the incidence of postoperative nausea and vomiting in the CPVB group compared with those in the CWI group (1/40 vs. 11/40, *P*=0.002). The length of ICU stay, time of chest tube removal, time of tracheal tube removal, and length of hospital stay were comparable between the two groups (Table 6).

## 7. Discussion

Our study revealed that CPVB can significantly reduce the chronic pain incidence and intensity at 3, 6, and 9 months after surgery and provide better acute pain relief up to 7 days after surgery when compared to local anesthetic continuous infusion but had no effect on long-term quality of life in patients with lung cancer. Our results may still promote the innovation of the postoperative analgesia mode of thoracic surgery and provide patients with better analgesia and faster rehabilitation quality after surgery.

The complexity of postoperative pain can be caused by a variety of factors, including the operative wound, muscle splitting, chest tube stimulation, and visceral pain; the complexity of pain generators necessitates multimodal postoperative analgesia [14]. For the control of postoperative pain, ultrasound-guided PVB is considered to be an alternative to TEA, which is regarded as the gold standard for postoperative analgesia in thoracic surgery [15]. Judging by the NRS scores at rest and activity during the 1st week after thoracoscopic surgery, our study proved that CPVB provided better acute pain relief as well as reduced the consumption of remedial analgesics, as has been reported in literature [2]. PVB is a technique in which a local anesthetic is injected into the thoracic paravertebral space containing the intercostal nerves, the sympathetic chain, and the dorsal rami of the spinal nerves. Therefore, PVB can produce ipsilateral sensory, motor, and sympathetic nerve blockade [16]. The injected local anesthetic may diffuse upward and downward along the paravertebral space. A single injection could produce anesthesia at 4-5 spinal levels, covering a wide plane [17]. In contrast, the local anesthetic continuous infusion of a wound cannot produce such a broad analgesic plane, which may be the reason why CPVB is superior to local anesthetic continuous infusion of a wound. In addition, the study found differences in the Barthel ADL scores on the 1st, 2nd, 3rd, and 7th postoperative days between the two groups. This may be related to the patients' perception of pain. Patients in the CPVB group experienced lower postoperative pain, which increased their ability to perform autonomous activities and accelerated their postoperative recovery.

Several studies have demonstrated that acute pain intensity is the strongest predictor of chronic pain [18–21]. In 1994, Katz et al. first showed that acute pain intensity within 24 hours postoperatively predicted chronic pain at 1.5 years after the operation in a prospective study [18]. Kampe et al. also reported that higher pain scores during the first five postoperative days seem to be the strongest risk factors for the development of chronic pain [19]. Our study also showed that the management of acute pain has a significant effect on the development of chronic pain. Patients with more severe acute pain (NRS ≥ 2 on the first postoperative day) were more likely to experience chronic pain. Acute pain in the early stages after surgery is thought to cause neuroplastic changes in the dorsal horn of the spinal cord, while pain can also cause sensitivity of the central nervous system and abnormalities in neurotransmitters among neurons, transforming acute postoperative pain into CPSP [22]. Strengthening the management of acute pain is of great significance to prevent the occurrence of CPSP. The highly effective analgesia provided by CPVB not only prevented acute pain but also provided afferent nerve block for several days after the operation to ensure that neuroplasticity was prevented, thereby avoiding the occurrence of chronic pain to a significant extent [23].

The reported prevalence of chronic pain after thoracic surgery is quite variable, ranging from 14.3 to 90% [24–26]. Our findings that show almost 20% of patients in the CPVB group and 42.5% in the CWI group were still reporting pain at 3 months postoperatively and that 7.5% of patients in the CPVB group and 25% of patients in the CWI group were still in pain at 9 months are consistent with the reported literature [25, 26]. In both groups, the chronic pain incidence decreased with time, and there were significant differences in the incidence of CPSP at 3, 6, and 9 months after the operation. In addition, at 3, 6, and 9 months postoperatively, the impact scores for the effects of pain on daily life and treatment measures were significantly decreased in the CPVB group compared with those in the CWI group. It is suggested that CPVB can not only reduce the incidence of CPSP but also reduce the intensity of CPSP. This is similar to the findings in previous studies. A meta-analysis showed that PVB can effectively prevent chronic pain after breast surgery, reducing the incidence of such a pain [27]. Borys et al. reported that patients who received PVB had a lower CPSP incidence and experienced a lower CPSP intensity at 6 months postoperatively than did control patients and patients receiving ketamine administration [24]. The explanation for the lower incidence of chronic pain in the PVB groups in these studies may be that PVB, which can not only produce a very dense afferent blockade of sensory information but also completely block transmission within the sympathetic chain, reduces central sensitization, thereby leading to less CPSP [28, 29].

Severe persistent postsurgical pain mandates the liberal use of painkillers, including opiates, which itself increases the risk of chronic pain emergence [30]. The related mechanisms may be extensive tissue damage due to opiate use and opioid-induced hyperalgesia after prolonged and high-dose use of opioids [31]. The results of the present study suggest that patients in the CPVB group used less rescue analgesia and exhibited less postoperative opioid use, which was also one of the reasons why the incidence of CPSP in the CPVB group was lower than that in the CWI group. There was a significant reduction in the incidence of postoperative nausea and vomiting in the CPVB group compared with that in the CWI group (1/40 vs. 11/40, *P*=0.002), which may be related to the amount of rescue analgesics used.

To evaluate the long-term postoperative quality of life of patients with lung cancer undergoing thoracoscopic surgery, SF-12 was selected as the evaluation tool in this study. SF-12 was developed and validated as a generic short-form instrument for measuring health-related quality of life. The tool has been widely applied to assess important quality of life domains in medical studies [32]. In the present study, the two groups were comparable regarding SF-12 scores. It is suggested that compared with continuous local-anesthetic infusion into wounds, continuous PVB does not affect the long-term quality of life of lung cancer patients undergoing thoracoscopic surgery. This was comparable to the findings of the Chiu et al. trial, in which it was found that the quality of life at 1 year after breast cancer surgery was similar in the two groups as assessed by SF-12 [33]. Our study did not determine the baseline quality of life for all patients. Therefore, it could not be concluded whether CPSP impaired the patients' quality of life.

### 7.1. Limitations

Some limitations of this study need to be considered. First, the sample size of our study (a single-center investigation) is slightly small, which may have biased the research results. Second, there was no blank control group in our clinical study because our local ethics committee believes that it is immoral to use normal saline for PVB or wound infiltration. Third, we failed to assess the nature of the postoperative chronic pain, that is, whether it was purely nociceptive pain or neuropathic pain. Fourth, all subjects in this study were given a 15 ml anesthetic load dose when they received paravertebral nerve block, but there was no significant difference in BMI between the two groups, so the average local anesthetic dose received by patients did not differ between the groups. Fifth, the paravertebral nerve block and catheter placement were performed after surgery, primarily to match the timing of analgesia in the CWI group and to minimize bias between groups due to the different timing of analgesia. Sixth, we did not collect more information about the patients' environment and behavior, which may have influenced the development of chronic pain. As a result, intraoperative drug infusion into the paravertebral space was not considered. However, despite such flaws, this study may still provide new insights into pain management in patients undergoing thoracic surgery.

## 8. Conclusion

Ultrasound-guided CPVB can reduce the number of patients receiving remedial analgesics after thoracoscopic surgery, promote the quality of early postoperative rehabilitation, and reduce the incidence and intensity of CPSP within 9 months after thoracoscopic surgery by better acute pain control, but there was no significant advantage in the long-term quality of life within 9 months postoperatively.

## Figures and Tables

**Table 1:** Patient characteristics and operation details.

	CPVB (*n* = 40)	CWI (*n* = 40)	*P* value
General information			
Age (y)	53.50 (47.00–58.75)	60.50 (51.00–65.00)	0.060
Gender (male/female)	12/28	16/24	0.348
BMI (kg/m^2^)	23.85 ± 2.51	23.17 ± 2.87	0.262
ASA classification (II/III)	30/10	31/9	0.793
Comorbidities			
Smoking history (*n*, %)	8 (20)	12 (30)	0.302
Drinking history (*n*, %)	9 (22.50)	8 (20)	0.785
Coronary heart disease history (*n*, %)	1 (2.50)	1 (2.50)	1.000
Hypertension history (*n*, %)	8 (20)	7 (17.50)	0.775
Diabetes history (*n*, %)	6 (15)	2 (5)	0.136
Baseline laboratory values			
Preoperative hemoglobin (g/L)	137.50 (128.25–149.75)	134.00 (130.00–145.00)	0.828
Preoperative white blood cell (10^9^/L)	5.82 (4.76–6.52)	5.22 (4.52–6.23)	0.405
Preoperative blood glucose (mmol/l)	5.70 (5.13–6.30)	5.20 (5.00–6.00)	0.050
Intraoperative information			
Duration of the operation (min)	121.43 ± 33.64	114.60 ± 42.66	0.429
Duration of anesthesia (min)	167.88 ± 31.98	159.48 ± 47.74	0.358
Propofol dosage (mg)	430 (400–500)	475 (400–500)	0.351
Sufentanil dosage (*μ*g)	50 (40–50)	50 (45–50)	0.242
Remifentanil dosage (mg)	1.00 (1.00–1.40)	1.00 (0.93–1.50)	0.771
Infusion volume (ml)	1100 (1100–1100)	1100 (1100–1450)	0.172

Continuous variables with a normal distribution are presented as the mean ± standard deviation (SD), nonparametric variables are reported as the median (interquartile range (IQR)), and categorical variables are presented as *n* (%). CPVB, continuous paravertebral block; CWI, continuous wound infiltration; ASA, American Society of Anesthesiologists; BMI: body mass index.

**Table 2:** The intensity of CPSP at 3, 6, and 9 months postoperatively.

Groups	Time	The effect of the pain score on daily life					The level of pain treatment measures				
		A	B	C	D	*P*value	A	B	C	D	*P*value
CPVB	3^rd^ month	37	2	1	0	0.021	38	1	1	0	0.014
CWI		29	8	3	0		30	7	3	0	

CPVB	6^th^ month	38	1	1	0	0.048	39	0	1	0	0.030
CWI		32	6	2	0		34	4	2	0	

CPVB	9^th^ month	39	1	0	0	0.048	40	0	0	0	0.041
CWI		34	5	1	0		37	2	1	0	

Categorical variables are presented as *n* (%). CPVB, continuous paravertebral block; CWI, continuous wound infiltration.

**Table 3:** Barthel Activity of Daily Living (ADL) score at postoperative days 1–7.

	CPVB (*n* = 40)	CWI (*n* = 40)	*P* value
The Barthel ADL scores			
1^st^ day	75.00 (65.00–80.00)	67.50 (60.00–70.00)	0.002
2^nd^ day	90.00 (80.00–95.00)	75.00 (70.00–90.00)	<0.001
3^rd^ day	95.00 (90.00–95.00)	90.00 (85.00–95.00)	0.005
7^th^ day	95.00 (95.00–100.00)	95.00 (95.00–100.00)	0.379

Categorical variables are presented as *n* (%). CPVB, continuous paravertebral block; CWI, continuous wound infiltration; ADL, activities of daily living.

**Table 4:** Long-term quality of the life score at 3, 6, and 9 months postoperatively.

	CPVB (*n* = 40)	CWI (*n* = 40)	*P* value
SF-12 PCS			
3^rd^ month	21.31 ± 1.30	20.83 ± 1.41	0.348
6^th^ month	23.42 ± 1.41	22.59 ± 2.13	0.262
9^th^ month	23.97 ± 1.74	23.62 ± 2.15	0.322
SF-12 MCS			
3^rd^ month	23.43 ± 2.24	22.51 ± 2.31	0.210
6^th^ month	24.51 ± 2.53	24.35 ± 2.55	0.168
9^th^ month	24.63 ± 3.13	23.78 ± 3.16	0.172

Continuous variables with a normal distribution are presented as the mean ± standard deviation (SD). CPVB, continuous paravertebral block; CWI, continuous wound infiltration; SF-12, 12-item Short-Form Health Survey; PCS, physical health summary; MCS, mental health summary.

**Table 5:** The relationship between patients with NRS-R ≥ 2 on the first postoperative day and the incidence of chronic pain at 3, 6, and 9 months postoperatively.

	NRS-R ≥ 2	NRS-R < 2	*P* value
POD 1	48	32	
3^rd^ month	20 (41.67)	5 (15.63)	0.014
6^th^ month	16 (33.33)	4 (12.50)	0.035
9^th^ month	11 (22.92)	2 (6.25)	0.048

Categorical variables are presented as *n* (%). CPSP, chronic postsurgical pain; NRS-R, numerical rating scale at rest; POD, postoperative day.

**Table 6:** Postoperative-related data.

	CPVB (*n* = 40)	CWI (*n* = 40)	*P* value
Rescue analgesia	2 (5.00)	13 (32.50)	0.002
Analgesia satisfaction	33 (82.50)	15 (37.50)	<0.001
Postoperative nausea and vomiting	1 (2.50)	11 (27.50)	0.002
Respiratory depression	0	2 (4.80)	0.155
Retention of urine	0	1 (2.50)	0.317
Local bleeding puncture site hematoma	0	0	1.000
Local anesthetic poisoning	0	0	1.000
Length of stay in ICU (h)	12.96 ± 6.12	15.48 ± 6.61	0.082
Time of tracheal tube removal (h)	1.49 ± 0.48	1.59 ± 0.62	0.413
Time of chest tube removal (d)	3.10 ± 1.41	3.43 ± 1.80	0.371
Length of hospitalization (d)	4.13 ± 1.59	4.23 ± 1.89	0.798

CPVB, continuous paravertebral block; CWI, continuous wound infiltration. Categorical variables are presented as *n* (%).

## Data Availability

The datasets generated and/or analyzed during the current study are not publicly accessible but are available from the corresponding author upon reasonable request.
